# Dentin matrix protein 1 and HUVEC-ECM scaffold promote the differentiation of human dental pulp stem cells into endothelial lineage: implications in regenerative medicine

**DOI:** 10.3389/fphys.2024.1429247

**Published:** 2024-07-08

**Authors:** Amudha Ganapathy, Karthikeyan Narayanan, Yinghua Chen, Cassandra Villani, Anne George

**Affiliations:** Department of Oral Biology, University of Illinois Chicago, Chicago, IL, United States

**Keywords:** dental pulp stem cells, dentin matrix protein 1, human vascular endothelial cells, endothelial cells, endothelial cell differentiation, angiogenesis, tissue regeneration

## Abstract

Reprograming of the dental pulp somatic cells to endothelial cells is an attractive strategy for generation of new blood vessels. For tissue regeneration, vascularization of engineered constructs is crucial to improve repair mechanisms. In this study, we show that dentin matrix protein 1 (DMP1) and HUVEC-ECM scaffold enhances the differentiation potential of dental pulp stem cells (DPSCs) to an endothelial phenotype. Our results show that the differentiated DPSCs expressed endothelial markers CD31 and VE-Cadherin (CD144) at 7 and 14 days. Expression of CD31 and VE-Cadherin (CD144) were also confirmed by immunofluorescence. Furthermore, flow cytometry analysis revealed a steady increase in CD31 and VE-Cadherin (CD144) positive cells with DMP1 treatment when compared with control. In addition, integrins specific for endothelial cells were highly expressed during the differentiation process. The endothelial cell signature of differentiated DPSCs were additionally characterized for key endothelial cell markers using gene expression by RT-PCR, Western blotting, immunostaining, and RNA-seq analysis. Furthermore, the angiogenic phenotype was confirmed by tubule and capillary sprout formation. Overall, stimulation of DPSCs by DMP1 and use of HUVEC-ECM scaffold promoted their differentiation into phenotypically, transcriptionally, and functionally differentiated bonafide endothelial cells. This study is novel, physiologically relevant and different from conventional strategies.

## Introduction

Angiogenesis is a crucial physiological process for wound healing, tissue repair, and regeneration. (DiPietro, 2016; Shi et al., 2023). During bone development and regeneration, the growth of blood vessels are coupled with osteogenesis (Boston et al., 2021; Schott et al., 2021). Blood vessels are important for the transport of nutrients, oxygen, ions, circulating cells and facilitate the removal of metabolic waste (Rademakers et al., 2019). They also provide instructive signals for organogenesis. The growing body of evidence indicates that the absence of a functional and appropriate vascular network may result in necrosis and impaired bone formation (Lee et al., 2021; De Silva et al., 2023).

Human dental pulp stem cells (hDPSCs) are neural crest derived adult stem cells with multi-differentiation potential (Janebodin et al., 2011). DPSCs show remarkable versatility and respond to extracellular cues by altering their phenotype (Kim et al., 2012). Several studies have shown that DPSCs have the potential to favor angiogenesis via paracrine mechanisms (Mattei et al., 2021; Delle Monache et al., 2022). Therefore, DPSCs would be an ideal cell source for generating vasculogenic endothelial cells (ECs).

The extracellular matrix (ECM) provides physical and mechanical support to the embedded cells. Earlier reports shows that tissue specific ECM signature provides instructional cues for organogenesis, cellular differentiation, migration, wound healing, immune response, and tissue remodeling (Xia et al., 2018; Kim et al., 2023). Specifically, ECM bound functional biomacromolecules (e.g., growth factors) provide spatial, chemical, and functional cues (Lutolf and Hubbell, 2005). Published reports show the influence of ECM on stem cell differentiation (Novoseletskaya et al., 2019). Therefore, recent engineering strategies have focused on mimicking the ECM composition of ECs to stimulate angiogenesis and tissue regeneration (Pina et al., 2019; Xie et al., 2021).

ECs secrete specific matrix macromolecules, which play a vital role in enabling the differentiation of stem cells into ECs (Davis and Senger, 2005). Notably, the ECM derived from ECs consists of isoforms of collagen type IV, laminin, heparan sulfate, proteoglycans (and nidogen-1/nidogen-2 (Witjas et al., 2019). These components serve as both structural and signaling proteins, as well as facilitating cell attachment and viability. Cell-cell and cell-matrix adhesions are mediated by cadherins and integrins and are important players in the development of ECs. Both these adhesion receptors have been implicated in cell survival, cytoskeletal organization and cell migration (Aman and Margadant, 2023). VE-cadherin expression is tissue-specific and exclusive to ECs. In addition to its adhesive functions, VE-cadherin regulates various cellular processes such as cell proliferation and apoptosis and modulates vascular endothelial growth factor receptor function, therefore, VE-cadherin is essential during embryonic angiogenesis (Vestweber, 2008).

ECM from several types of ECs have been successfully used to differentiate human mesenchymal stem cells into endothelial lineage cells in 2D or 3D culture settings (Gong et al., 2017). Furthermore, stem cells derived from human exfoliated deciduous teeth, when cultured on the ECM of HUVECs for 7 days, expressed CD31 and VEGFR2. Additionally, these cells were functionally assessed through a tube formation assay on Matrigel (Gong et al., 2017; Xu et al., 2019).

Dentin matrix protein 1 (DMP1) is a bone and tooth-specific non-collagenous ECM protein initially identified from the dentin matrix. DMP1 is highly anionic and rich in aspartic acid, glutamic acid and serine residues (George and Veis, 2008). Apart from its role in matrix mineralization (Hao et al., 2004), DMP1 can function as a signaling molecule impacting osteoblast and odontoblast differentiation at several stages of development (Ravindran and George, 2014). DMP1 is an essential factor in the terminal differentiation of ectomesenchyme-derived neural crest cells into functional odontoblasts (Narayanan et al., 2001). Extracellular DMP1 is endocytosed via the cell surface receptor GRP78 (Ravindran et al., 2008) and triggers calcium mediated signaling events resulting in the differentiation of preosteoblasts to osteoblasts (Eapen et al., 2010).

In the current study, we show that, DMP1 enhances the differentiation of DPSCs cultured on HUVEC-ECM scaffold towards an EC phenotype. The EC signature of differentiated DPSC cells were characterized using gene expression by RT-PCR, immunostaining, and RNA sequencing analysis. Furthermore, the functional assessment of the differentiated cells was conducted using tube formation and sprout assay. Understanding the role of vasculogenic ECM and the influence of DMP1 as a signaling molecule on DPSCs transformation into ECs is key to the field of regenerative vascular medicine.

## Materials and methods

### Cell culture

Human Dental pulp Stem Cells (hDPSCs), as provided by Dr. Shi from the University of Pennsylvania, were cultured in α-minimum Eagle’s medium from Thermo Fisher Scientific (Waltham, MA). This medium was supplemented with 20% fetal bovine serum (FBS) and 1% Antibiotic-Antimycotic (100X), both from Thermo Fisher Scientific. The cells were maintained at 37°C and 5% CO_2_. When the cells reached approximately 90% confluence, they were subcultured at a 1:3 ratio (passage). Cells from passages 3 to 6 were utilized in all experiments.

### HUVEC-ECM preparation

A non-enzymatic cell lysis procedure was employed to produce ECM from human umbilical vein endothelial cells (HUVEC-ECM) as described earlier (Narayanan et al., 2014). Briefly, HUVECs were seeded onto 6 well plates coated with 0.2% gelatin at a density of 0.3 × 10^6^ per well in EBM-2 media (EBM-2, Lonza, Morristown, NJ). After reaching 90% confluence, cells were decellularized with 20 mM ammonium hydroxide (Thermo Fisher Scientific) for 5 min, followed by a PBS wash to obtain HUVEC-ECM. The plates containing HUVEC-ECM were used immediately or stored at 4°C in PBS containing 1% antibiotic-antimycotic solution until further use.

### Differentiation of DPSCs on HUVEC-ECM

DPSCs were seeded at a density of 0.3 × 10^6^ cells per well onto plates coated with HUVEC-ECM and cultured in EBM-2 media supplemented with 10% FBS. When needed DMP1 (500 ng/mL) (Eapen et al., 2010) was added to the media. The cells were fed with fresh prepared media every other day.

### Immunofluorescence staining

DPSC cells were cultured on a HUVEC-ECM coated cover glass for 7 and 14 days, with and without DMP1 stimulation. The cells were then fixed for 1 hour at 4°C in 10% neutral formalin. The cells were rinsed with PBS and permeabilized with 0.5% Triton X-100 in PBS for 10 min at room temperature. Subsequently, the cells underwent blocking with 5% BSA in PBS for 1 h. Specific primary antibodies against CD31 and VE-Cadherin (CD144) (ab28364, ab33168, Abcam, Cambridge, MA), vWF, GRP78 (sc-365712, sc-166490, Santa Cruz Biotechnology, Dallas, TX) and VEGR2 (rabbit, #2479, Cell Signaling Technology) were added and incubated overnight at 4°C. Fluorescent secondary antibodies (Thermo Fisher Scientific), goat anti-rabbit Alexa Fluor 594, goat anti-mouse Alexa Fluor 488 were added for 2 h at room temperature. The cover glass was attached to a glass slide using a Mounting Medium with DAPI (Vector Laboratories, Inc., Newark, CA). Following this, the cells were imaged using a Zeiss 710 Meta Confocal Microscope at the UIC RRC Facility.

### Real-time quantitative PCR

DPSCs stimulated with and without DMP1were cultured on HUVEC ECM plates. Total RNA was isolated using Trizol reagent (Invitrogen, Waltham, MA) according to the manufacturer’s protocol. The purity (A260/A280) and concentration of RNA were determined using a NanoDrop 2000 spectrophotometer (Thermo Scientific, Waltham, MA, United States). Using the Maxima First Strand cDNA Synthesis Kit with dsDNase (Thermo Scientific, Waltham, United States), reverse transcription of cDNA was carried out in accordance with the manufacturer’s instructions. The FastStart Universal SYBR green master reagent (Roche diagnostics, Indianapolis, IN, United States) was used for RT-qPCR utilizing ABI StepOnePlus instrument (Thermo Fisher Scientific). The gene expression levels were estimated by the 2^−ΔΔCT^ method with the *GAPDH* gene expression level as an internal control. Oligo DNA primers were synthesized by IDT (Integrated DNA Technologies, Inc.) and are listed in Table 1.

**TABLE 1:** Primers for 8 upregulated genes involved in endothelial differentiation and housekeeping gene GAPDH as an endogenous reference.

*Genes*	*Forward Primer (5′-3′)*	*Reverse Primer (5′-3′)*
*CD31*	*CTG CCA GTC CGA AAA TGG AAC*	*CTT CAT CCA CCG GGG CTA TC*
*VE-Cadherin*	*TTG GAA CCA GAT GCA CAT TGA T*	*TCT TGC GAC TCA CGC TTG AC*
*vWF*	*TGC AAC ACT TGT GTC TGT CG*	*CGA AAG GTC CCA GGG TTA CT*
*VEGFA*	*AGG GCA GAA TCA TCA CGA AGT*	*AGG GTC TCG ATT GGA TGG CA*
*ITGA6*	*TTG AAT ATA CTG CTA ACC CCG*	*TCG AAA CTG AAC TCT TGA GGA*
*ITGAV*	*AAT CTT CCA ATT GAG GAT ATC AC*	*AAA ACA GCC AGT AGC AAC AAT*
*ITGB3*	*CCG TGA CGA GAT TGA GTC A*	*AGG ATG GAC TTT CCA CTA GAA*
*ITGB4*	*AGA CGA GAT GTT CAG GGA CC*	*CCT CTC CTC TGT GAT TTG GAA*
*GAPDH*	*GGA GCG AGA TCC CTC CAA AAT*	*GGC TGT TGT CAT ACT TCT CAT GG*

### Functional assessment of differentiated DPSCs

#### Tube formation assay

The capability of differentiated DPSCs to form tubule-like structures was determined using a tube formation assay. DPSCs stimulated with and without DMP1 were first cultured for 7 days. The cells were collected using Accutase (Thermo Fisher Scientific) and centrifuged for 200 g for 5 min. The control and differentiated DPSCs (2 × 10^4^) were seeded in the solidified Matrigel (50 μL, BD Bioscience, San Jose, CA) in a 96-well plate and incubated at 37°C. The formation of tubular structures was examined, and images were obtained at different time points (2, 4, and 24 h) using a light microscope (EVOS, Thermo Fisher Scientific). Measurements of tube length, number of branches, number of loops, and average loop area were quantitatively measured using “Tube formation FastTrack AI Image Analysis” (MetaVi Labs Inc., Germany).

#### Spheroid sprouting assay

Spheroid sprouting assay was performed to investigate the functional ability of DPSC-derived ECs according to published protocol (Heiss et al., 2015). 25 µL of suspension containing differentiated DPSCs (∼1000 cells) in α-MEM containing 20% Methylcellulose (Sigma-Aldrich, ST. Louis, MO) was pipetted onto petri-dishes (Fisher Scientific). Spheroids were formed via hanging-drop method for 24 h at 37°C. The spheroids (∼35) were collected and centrifuged at 200g for 5 min, re-suspended in EBM-2 media with methylcellulose (0.5% w/v) supplemented with 20% FBS and kept on ice until used. In a separate tube, spheroid mixture containing rat tail collagen type I (3.5 mg/mL, Thermo Fisher Scientific, Waltham, MA), Medium 199 (Thermo Fisher Scientific, Waltham, MA, and NaOH (1 M) in a ratio of 8:1:1 respectively was prepared on ice. Following this, 1 mL of the spheroid mixture (∼20 spheroids, prepared with equal volume of collagen solution and spheroid solution) was transferred to a pre-warmed (37°C) 24-well plate. Upon polymerization of the mixture at 37°C for 30 min, 0.2 mL of EBM2 was added to each well and incubated at 37°C for 24 h. Images of ten spheroids per well were captured using a Zeiss microscope. The images were then analyzed for number and length of sprouts using ImageJ.

### Flow cytometry analysis and cell sorting

DPSCs were cultured on the HUVEC-ECM with or without DMP1. After 7 days of differentiation the cells were collected using Accutase (Thermo Fisher Scientific) and centrifuged at 300 g for 5 min, washed with PBS, and then treated with an antibody for 40 min at room temperature in the dark. The antibodies used were BD Pharmingen™ FITC Mouse Anti-Human CD31 (BD Biosciences, San Jose, CA), BD Pharmingen™ APC Mouse Anti-Human CD144 (BD Biosciences), and FITC or APC labelled isotype match IgG (BD Biosciences) as control. Subsequently, the cells were washed with PBS and suspended in Stain Buffer (BD Bioscience, San Jose, CA). For CD31 and VE-Cadherin (CD144) expression analysis, at least 20,000 cells were recorded using the CytoFLEX flow cytometry instrument at the UIC RRC facility and analyzed with FlowJo software (BD Biosciences). In addition, based on the expression levels of CD31 and VE-cadherin (CD144), flow cytometry sorting was conducted using a Moflo Astrios EQ device (UIC RRC facility). The pre-sorted and post-sorted DPSCs were used for RNA Seq-Analysis.

### RNA Seq-Analysis

Total RNA was extracted from post-sorted and pre-sorted DPSCs as described above, and from HUVECs serving as reference. RNAseq was performed by LC Sciences (Houston, TX). The genes counts obtained from bulk mRNA sequencing were imported to R/RStudio (R Core Team, 2024) for gene expression analyses. Ensembl count identifiers were converted to gene symbol annotation using the R package *org.Hs.eg.db* (Carlson, 2017). An EC marker list was obtained from PanglaoDB (Franzén et al., 2019) and uploaded to R/RStudio (R Core Team, 2024) and filtered to keep only genes that had a human specificity of 0.5 or greater, followed by Ensembl gene annotations for analysis. R package, *edgeR* (Robinson et al., 2010; McCarthy et al., 2012; Chen et al., 2016) was used for trimmed mean of means (TMM) normalization. One way ANOVA and Tukey HSD tests were performed on the normalized expression values using the *AICcmodavg* (Mazerolle et al., 2023) package. A gene is considered significantly differentially expressed if the adjusted *p*-value is less than 0.05% with fold change greater than 2. Heat maps of specified gene sets were plotted using the *ComplexHeatmap* (Gu et al., 2016; Gu, 2022) package. The Database for Annotation, Visualization, and Integrated Discovery (DAVID) (https://www.ncbi.nlm.nih.gov/pubmed/35325185) was employed to identify Gene Ontology pathways that were enriched in the sorted DMP1 treated groups.

### Western blot analysis

Differentiated DPSCs were harvested using a cell scraper, centrifuged for 15 min at 12,000 rpm and the cell pellets were lysed in RIPA buffer (Cell Signaling Technology, Danvers, MA) supplemented with 1% proteinase inhibitor cocktail (Millipore Sigma, Burlington, MA). The mixture was incubated on ice for 30 min with gentle shaking and was subsequently centrifuged at 14,200 rpm at 4°C for 30 min. The resulting supernatant was utilized as the total cellular proteins. The concentration was determined using Bio-Rad Protein Assay Dye Reagent Concentrate (Bio-Rad Laboratories, Hercules, CA) with BSA (Millipore Sigma) employed as a standard. Subsequently, 50 μg of the proteins were separated by electrophoresis on an SDS-PAGE gel and transferred to a PVDF membrane (Bio-Rad Laboratories) at 20 V, overnight. The membranes were probed with specific primary antibodies, secondary HRP-linked anti-IgG antibody, and visualized using ECL Western Blotting Substrate (Thermo Fisher Scientific) following the manufacturer’s instructions and described previously (Chen et al., 2021). Primary antibodies were: CD31 (Rabbit polyclonal, Abcam), VEGFA (Rabbit polyclonal, Abcam), VE-Cadherin (Rabbit mAb, Cell Signaling Technology), ITGB4 (Rabbit, Cell Signaling Technology), ITGB3 (Rabbit, Cell Signaling Technology), ITGA6 (Rabbit, Cell Signaling Technology), ITGAV (Rabbit, Cell Signaling Technology), ACTIN (Mouse mAb, Cell Signaling Technology).

### Statistical analysis

The data was reported as the mean and standard deviation derived from a minimum of 3 independent experiments. Statistical significance was assessed using the student’s t-test. Significance was attributed to *p*-values of ≤0.05 and ≤0.01.

## Results

### Characterization of HUVEC-ECM

**FIGURE 1:**
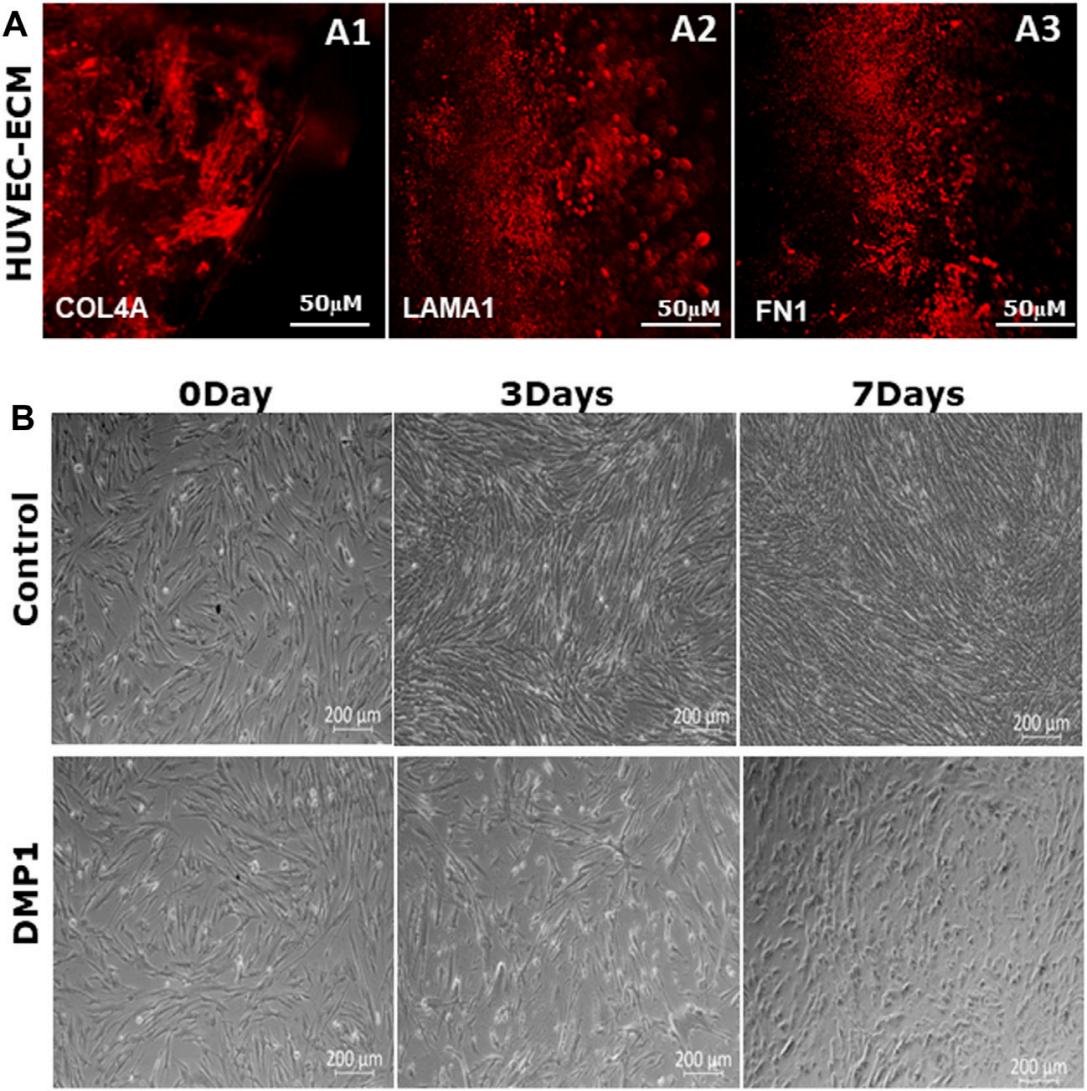
Characterization of decellularized HUVEC-ECM. **(A)** Representative images of immunofluorescence staining to demonstrate the presence of key HUVEC proteins in the ECM. ECM proteins shown are collagen type IV **(A1)**, laminin **(A2)** and fibronectin **(A3)**. **(B)** Representative phase-contrast microscopic images showing the morphology of DPSCs seeded on either HUVEC-ECM or HUVEC-ECM supplemented with DMP1 for 0, 3 and 7 days. Scale bar: 50 µm **(A)**; 200 µM **(B)**.

ECM extracted from HUVECs were isolated and characterized for key ECM proteins based on previously published studies (Kusuma et al., 2012). Immunohistochemical analysis revealed the presence of collagen IVA, fibronectin, and laminin 1 in the HUVEC-ECM (Figures 1A1–1A3). The absence of DAPI staining (data not shown) indicated specific staining for proteins in the ECM.

### Morphological changes of differentiated DPSC

DPSCs seeded on HUVEC-ECM exhibit distinct morphologies. DPSCs cultured on HUVEC-ECM for 3 and 7 days exhibited an elongated morphology with negligible number of spindle-shaped cells (Figure 1B, Control). In contrast, the addition of DMP1 and culturing for 3 days resulted in long spindle shaped cells which gradually transformed into smaller, spindle-shape cells at 7 days (Figure 1B, DMP1), indicating the influence of DMP1 on the differentiation of DPSCs into ECs cultured on HUVEC-ECM.

### Endothelial cell differentiation of DPSCs by HUVEC-ECM with DMP1

**FIGURE 2:**
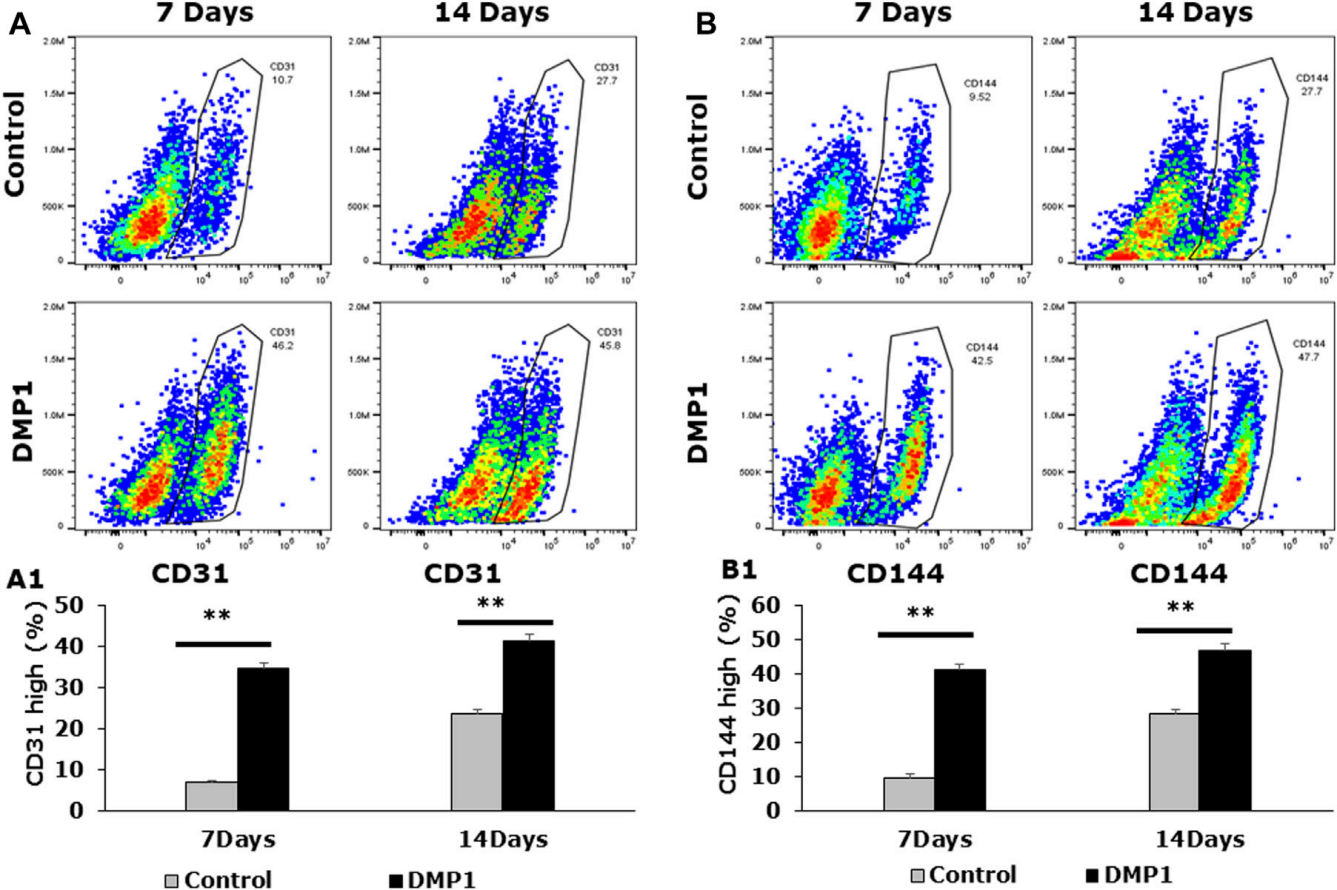
Detection of endothelial-specific surface markers in differentiated DPSCs. Flow cytometric analyses were carried out on differentiated DPSCs either for 7 or 14 days to evaluate the expression of CD31 **(A)** and VE-Cadherin (CD144) **(B)**. The flow cytometric profile of the differentiated DPSCs, either in the presence or absence of DMP1 displaying the fluorescence intensity on the x-axis and cell number on the y-axis, are presented. Mean and SD of CD31^+^ cells **(A1)** and CD144^+^ cells **(B1)** from three independent analyses are graphically represented. **: *p* < 0.01.

To demonstrate the differentiation of DPSCs into ECs under HUVEC-ECM with DMP1 conditions, flow cytometry analysis was performed by labeling the cells with CD31 FITC, CD144-APC (Figures 2A, B). Our results revealed that under HUVEC-ECM with DMP1 conditions, the CD31-FITC positive cell population of DPSCs was 34% at 7 days and 41% at 14 days (Figure 2A1), whereas under HUVEC-ECM alone, these percentages were notably lower at 7% and 24% for 7 and 14 days (Figure 2A1), respectively. Similarly, the CD144-APC positive cell population of DPSCs was substantially higher under HUVEC-ECM with DMP1 conditions compared to HUVEC-ECM alone, at 9% vs. 41% for 7 days and 28% vs. 47% for 14 days (Figure 2B1). These observations indicate that HUVEC-ECM contributes to the differentiation of DPSCs to endothelial-like cells; however, DMP1 synergistically enhanced the efficacy of the differentiation.

### Endothelial-related gene and protein expression of differentiated DPSCs

**FIGURE 3:**
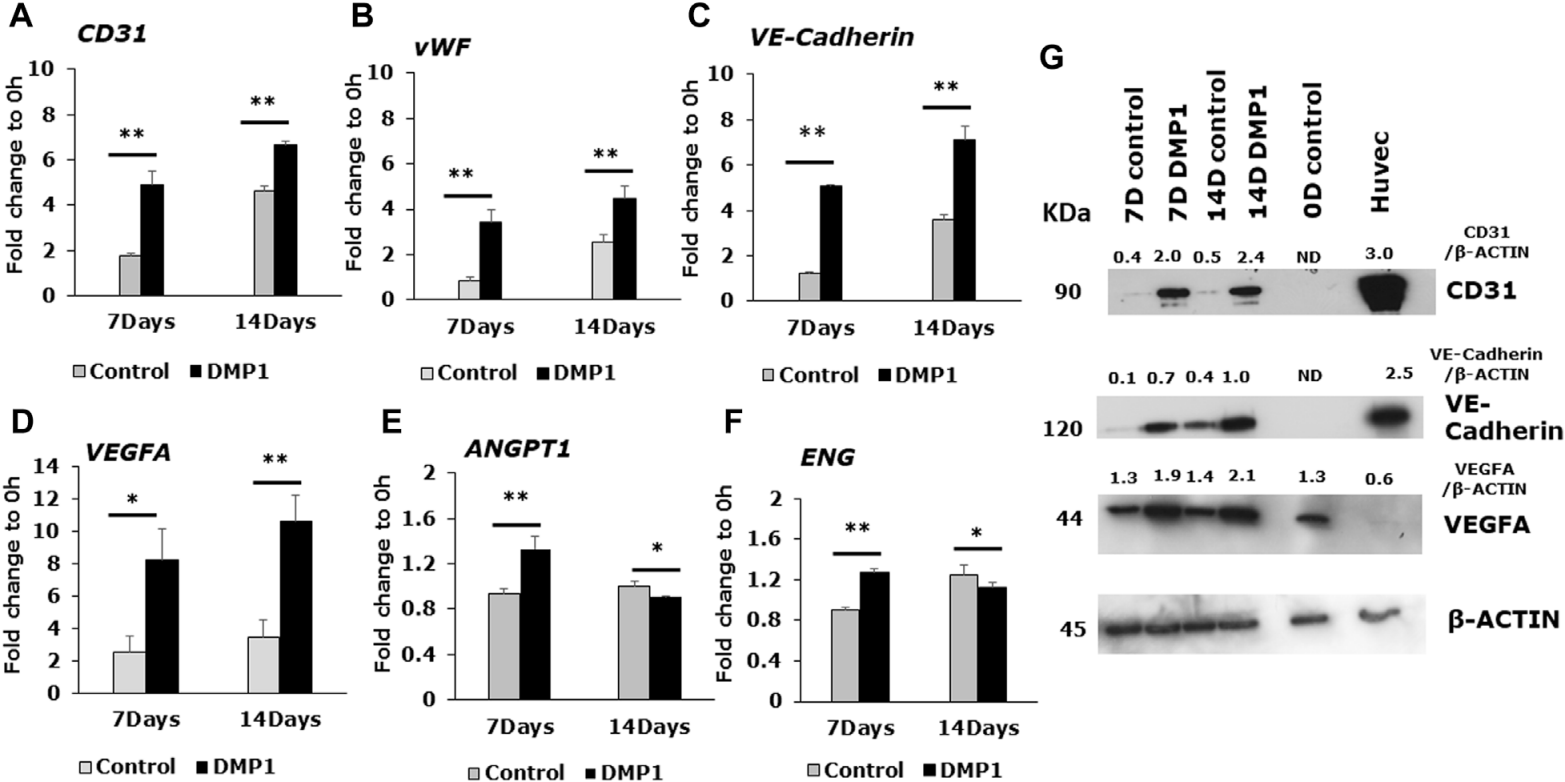
Gene expression analysis of angiogenic specific markers in the differentiated DPSCs. Quantitative RT-PCR was conducted using total RNA to evaluate the expression of key endothelial-specific genes. The bar graphs illustrate the fold changes with standard deviations in the expression of *CD31* **(A)**, *vWF* **(B)**, *VE-Cadherin* **(C)**, *VEGFA* **(D)**, *ANGPT1* **(E)**, and *ENG* **(F)** in DPSC cells cultured in HUVEC-ECM, both in the absence (Control, grey bar) and presence of DMP1 (DMP1, black bar) at 7 and 14 days. Fold changes were calculated with GAPDH as housekeeping gene as described in materials and methods section. *: *p* < 0.05 and **: *p* < 0.01. **(G)** Representative Western blots are shown for key endothelial proteins: VE-Cadherin, CD31 and VEGFA. β-ACTIN served as the internal control. Densitometric analysis of target proteins normalized to β-actin are shown.

**FIGURE 4:**
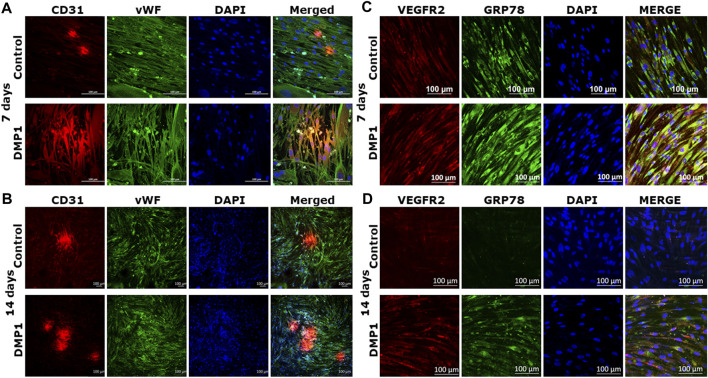
Immunostaining with endothelial markers in the differentiated DPSCs: CD31 and vWF expression of DPSCs differentiated either on HUVEC-ECM (Control), or HUVEC-ECM with DMP1 for 7 days **(A)** and 14 days **(B)** were detected by immunofluorescence. Similarly VEGFR2 **(C)** and GRP78 **(D)** expression levels were higher with DMP1 stimulation at 7 and 14 days. DAPI (blue) was used as nuclear stain. Scale bar = 100 µm.

DPSCs differentiated into ECs were further analyzed for endothelial cell signatures. Gene expressions for *CD31* and *vWF*, two well-known endothelial cell biomarkers were significantly higher in DPSCs differentiated in the presence of DMP1 (Figures 3A, B). Similarly, the expression of VE-Cadherin (CD144), a pivotal gene involved in endothelial cell junctions, showed a marked elevation in the presence of DMP1 (Figure 3C). The pro-angiogenic *VEGFA* was upregulated in differentiated DPSCs with DMP1 (Figure 3D), as well as *ANGPT1* to a lesser degree (Figure 3E). Finally, *ENG*, a component of the TGFβ receptor complex in ECs, were found to be slightly but significantly elevated in DPSCs treated with DMP1 (Figure 3F). Under the same differentiation conditions, the protein expression of CD31, VE-Cadherin (CD144), and VEGFA (Figure 3G) was elevated in DPSCs in the DMP1 treated group compared to the HUVEC-ECM alone as indicated by protein band densities normalized to β-ACTIN (density ratios indicated above target blot, Figure 3G.). This is further supported by the immunofluorescence analysis conducted at the 7 and 14-day time intervals (Figures 4A, B). Similarly, the expression of VEGFR2 and GRP78 increased with DMP1 stimulation at 7 and 14 days (Figures 4C, D).

### Integrin gene and protein expression in differentiated DPSC

**FIGURE 5:**
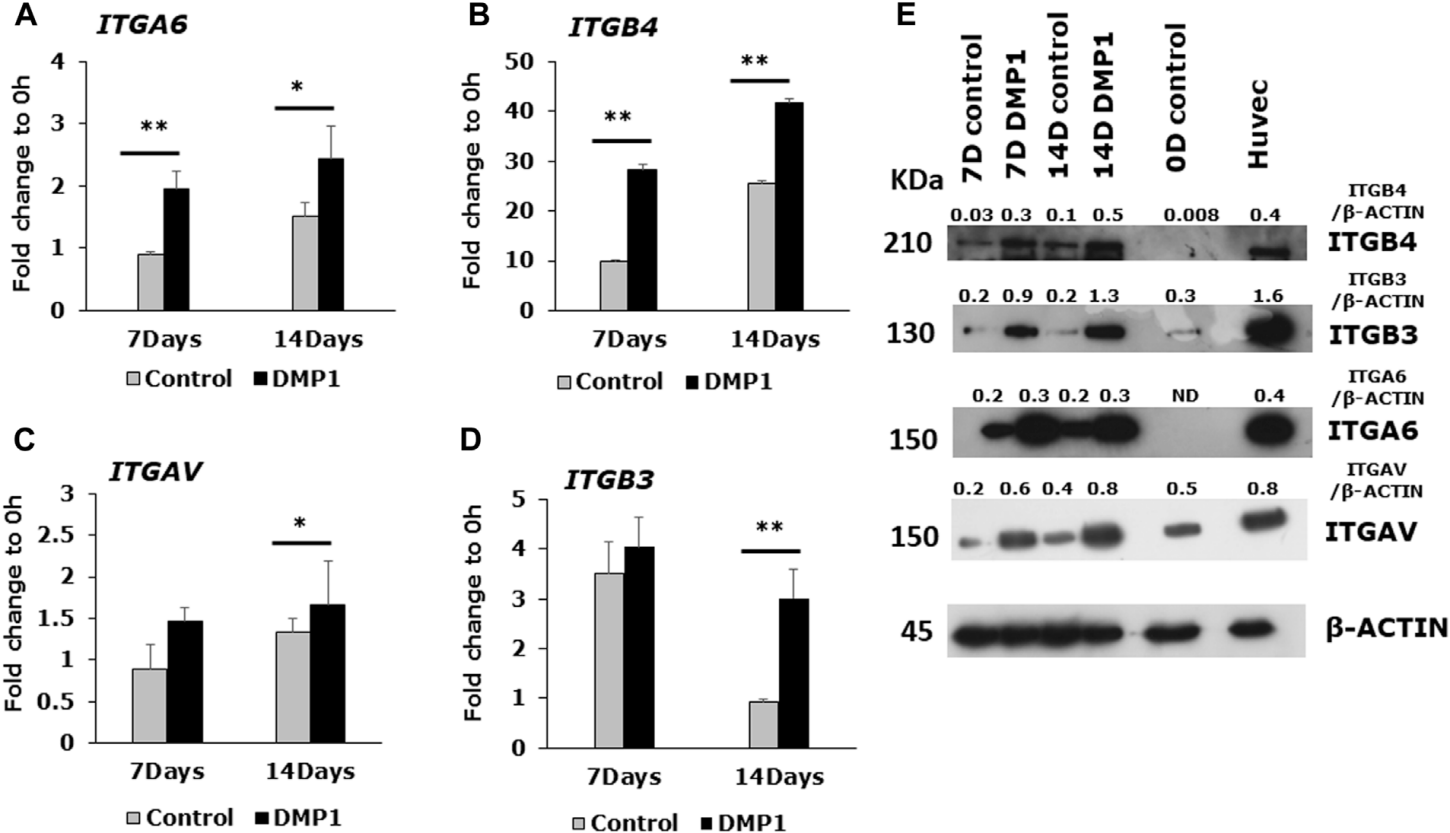
Endothelial cell specific integrin expression in DMP1 mediated differentiated DPSCs. DPSCs were cultured on HUVEC-ECM without (grey bars) or with DMP1 (black bars) for 7 and 14 days. The gene expression of integrin subunits α6 **(A)**, β4 **(B)**, αV **(C)**, β3 **(D)**, were assessed by quantitative RT-PCR. The mean fold change in expression with standard deviation are plotted, with GAPDH as the housekeeping gene. *: *p* < 0.05 and **: *p* < 0.01 indicate significance compared to the control. **(E)** Representative Western blots show the expression of integrin proteins (ITGB4, ITGB3, ITGA6 and ITGAV). β-actin served as the internal control. Densitometrical values of target proteins normalized to β-actin are shown.

Expressions of integrin family members were evaluated at both the mRNA and protein levels in DPSCs following differentiation under HUVEC-ECM conditions, with or without DMP1 (Figure 5). The expression of *ITGA6* (Figure 5A) and *ITGB4* (Figure 5B) exhibited significant increase at 7 and 14 days of differentiation of DPSCs with DMP1, in comparison to the control group. Similarly, the expression of *ITGAV* was significantly higher at 14 days (Figure 5C) while *ITGB3* (Figure 5D) was significantly higher at 7 and 14 days in the presence of DMP1 when compared to the control. Furthermore, Western blot analysis showed that DPSCs differentiated with DMP1 exhibited higher EC-specific protein expression at both 7 and 14 days compared to those cultured in the absence of DMP1 (Figure 5E).

### Endothelial cell-like phenotype of differentiated DPSCs

#### Matrigel-based tube formation assay

**FIGURE 6:**
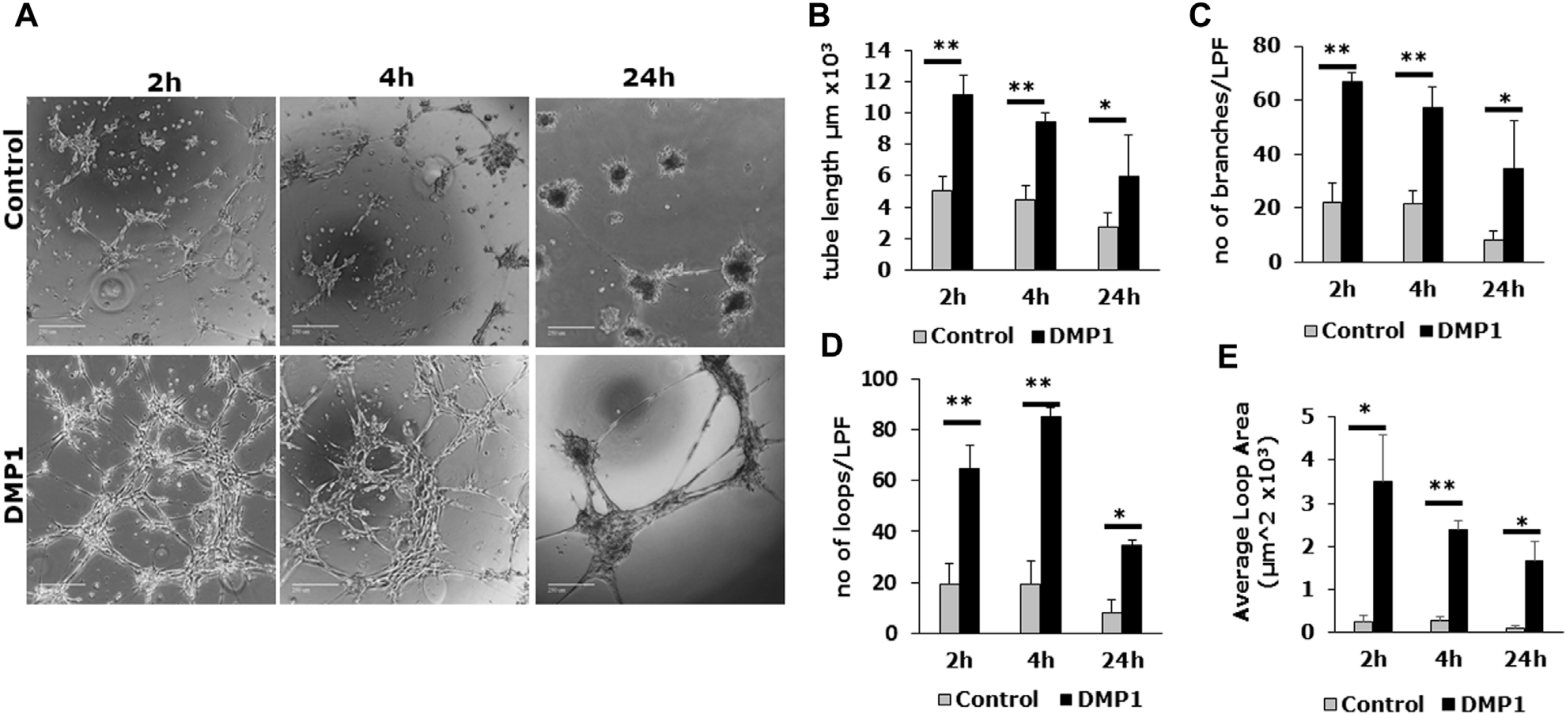
*In vitro* tubule formation assay: Differentiated DPSCs were assessed for tubule formation in Matrigel. **(A)** Representative images depicting the tubule-like network formed by the differentiated DPSCs, in the absence (Control) and presence of DMP1 (DMP1). Scale bar = 1000 µm. Measurements associated with tubule formation in the absence (Control, grey bars) and presence of DMP1 (DMP1, black bars): tube length **(B)**, number of branches **(C)**, number of loops **(D)** and average loop area **(E)**. Mean and SD from three measurements are presented. *: *p* < 0.05 and **: *p* < 0.01.

**FIGURE 7:**
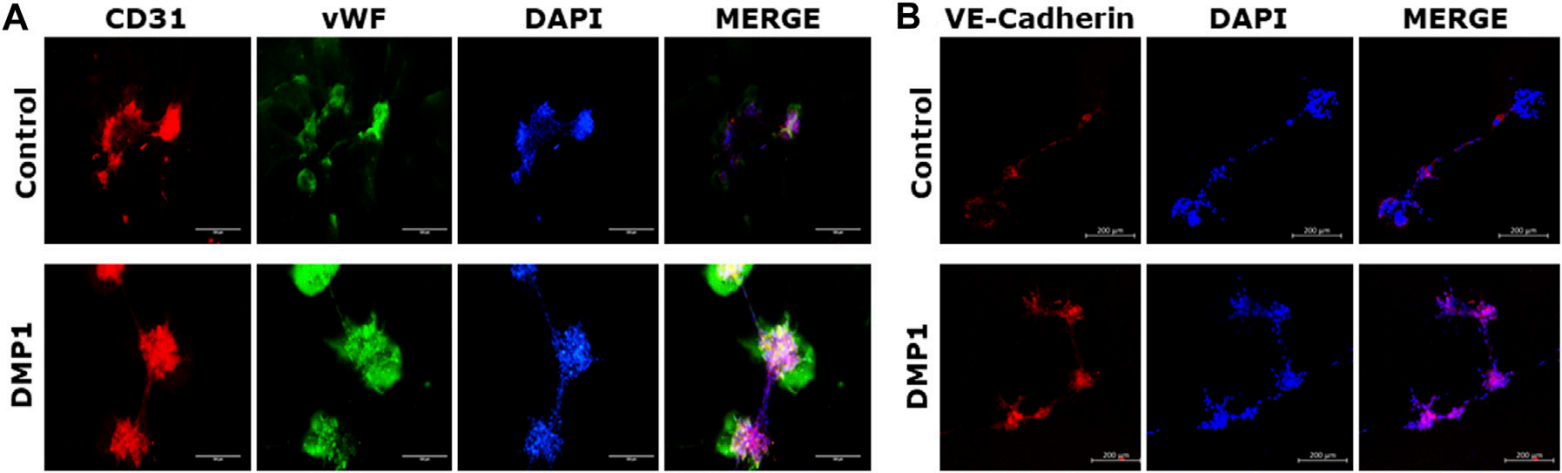
Immunostaining of tubules with endothelial specific markers. The tubule-like network formed on Matrigel by the differentiated DPSCs on HUVEC-ECM either in the absence (control) or in the presence of DMP1 were immunostained to detect endothelial markers. **(A)** immunostaining with CD31 (red) and vWF (green). **(B)** Immunostaing with VE-Cadherin (red). In both **(A,B),** DAPI (blue) represents nuclear staining. Scale bar = 100 µm, 200 µm.

DPSCs cultured on Matrigel coated with HUVEC-ECM in the presence of DMP1 exhibited adhesion, migration, and the formation of capillary-like tubules. These tubules underwent maturation into tubular networks within 2–24 h, demonstrating a significant enhancement in branching and vascular network tubule formation (Figure 6A, DMP1). In contrast, DPSCs cultured on HUVEC-ECM scaffold without DMP1 stimulation displayed cellular aggregates with sporadic network, followed by tubule aggregation at 24 h (Figure 6A, Control). Quantitative analysis indicated significant increase in tube characteristics, such as length (Figure 6B), branch numbers (Figure 6C), number of loops (Figure 6D), and average loop area (Figure 6E). The endothelial cell markers CD31 and vWF were prominently expressed in the tubes and nodules derived from DPSCs subjected to differentiation using HUVEC-ECM scaffold with DMP1 stimulation (Figure 7A, DMP1 vs. control). Furthermore, the expression of CD31 and vWF was exclusively evident in these cells. Additionally, there was an increase in the expression of VE-Cadherin (CD144) in the spheroids/sprouts derived from DPSCs that underwent differentiation with DMP1 (Figure 7B, DMP1 vs. control).

#### Spheroid *sprouting assay*

**FIGURE 8:**
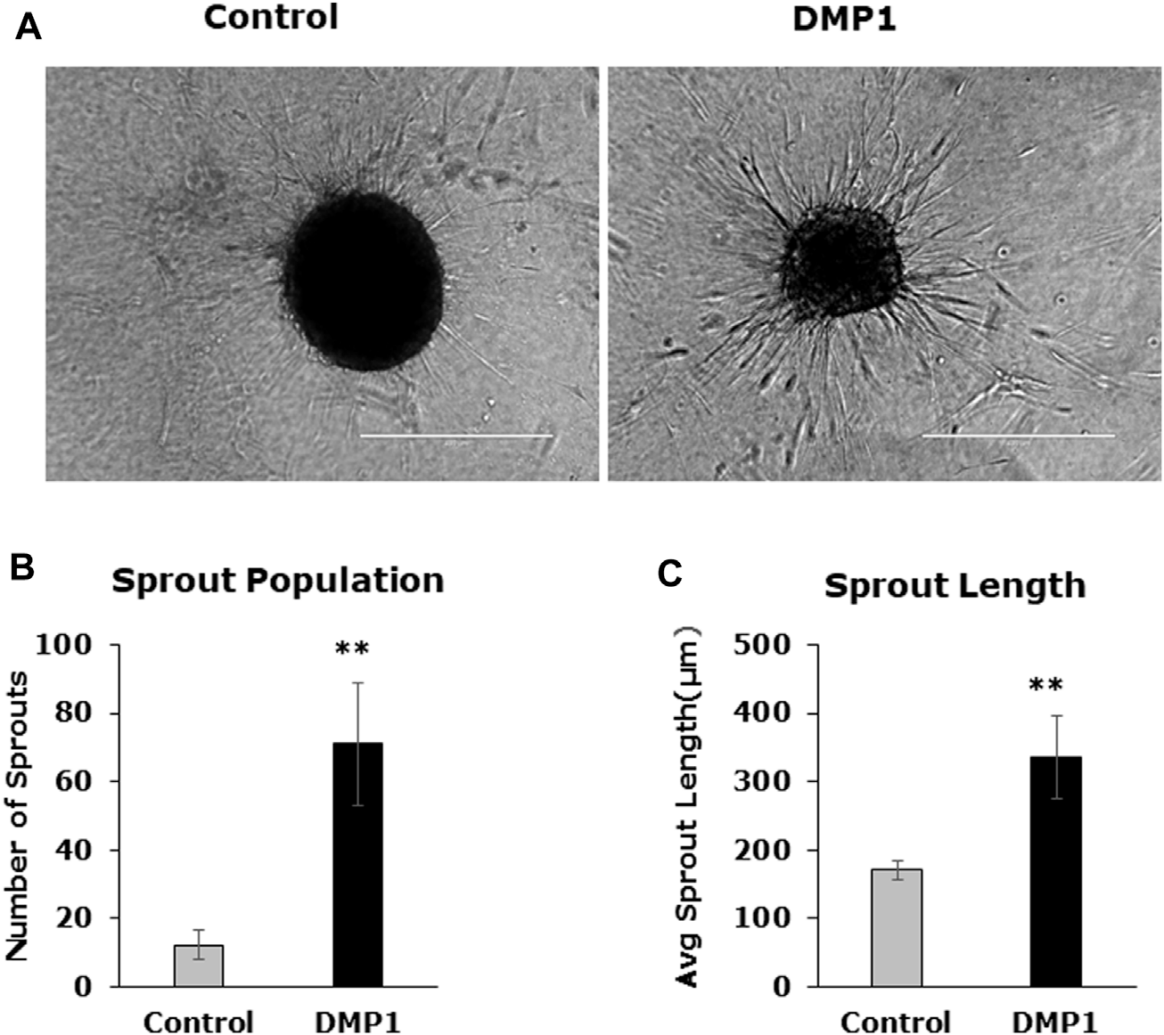
Spheroid sprouting assay. DPSC differentiated in the absence (Control) and presence of DMP1 (DMP1) were allowed to form spheroids by gravitation method. The spheroids were embedded in a collagen sandwich and observed under phase contrast microscope for sprouting after 24 h. Representative images are presented **(A)**. The parameters associated with sprouting such as sprout population **(B)**, and sprout length **(C)** of DPSCS derived EC cultured in HUVEC-ECM without (Control) and with DMP1 (DMP1) are shown. Scale bar = 100 µm.

Spheroids were generated using differentiated DPSCs under HUVEC-ECM, both with and without DMP1. Subsequently, they were embedded in a collagen type I scaffold to facilitate sprout formation, in accordance with the methods outlined. Following a 24-h incubation period, the sprouts originating from differentiated DPSCs under HUVEC-ECM with DMP1 demonstrated a notable increase in number (Figure 8B) and length (Figure 8C) when compared with those generated under HUVEC-ECM alone (Figure 8A, DMP1 vs. control).

### RNA seq analysis

**FIGURE 9:**
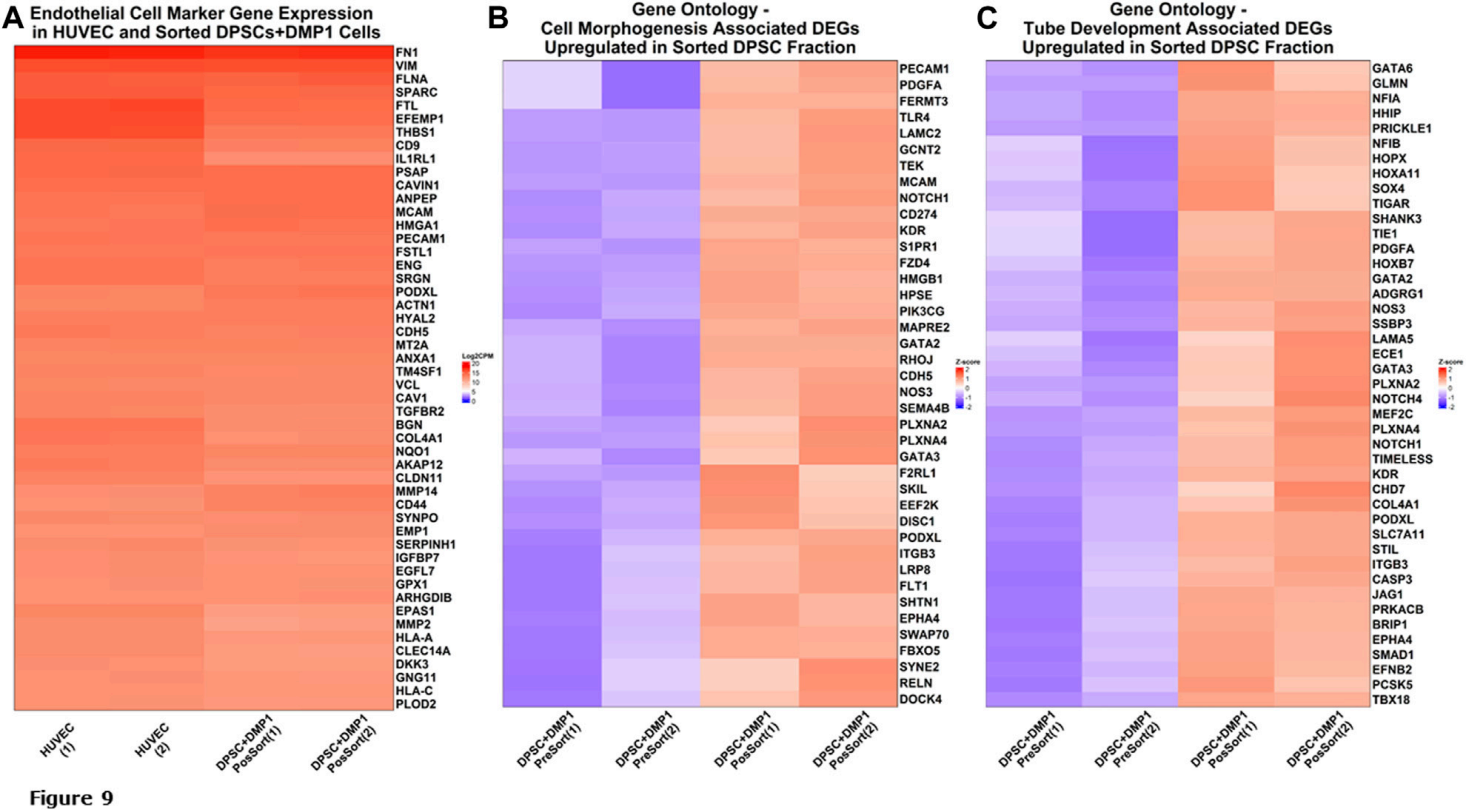
RNA Sequencing analysis. Gene Expression of Endothelial Cell Markers. **(A)** Heatmap of 50 endothelial cell marker genes with the highest expression in HUVECs are shown along with corresponding expression in the sorted fraction of DPSCs treated with DMP1. **(B)** Heatmap of differentially expressed genes upregulated in sorted fraction of DPSCs treated with DMP1 (red) compared to the unsorted DPSCs treated with DMP1 (blue) for the gene ontology cell morphogenesis pathway. **(C)** Heatmap of differentially expressed genes upregulated in sorted fraction of DPSCs treated with DMP1 (red) compared to the unsorted DPSCs treated with DMP1 (blue) for the gene ontology cell tube development pathway.

Of 985 endothelial cell markers, with a specificity in humans >0.05, 730 were present in RNA sequencing data for sorted DPSCs treated with DMP1 and HUVECs. Figure 9A shows 50 genes with the highest relative gene expression and demonstrates a lack of significant difference between transcriptomic profiles for the HUVECs and DMP1 treated DPSCs. This is supported by one-way ANOVA and Tukey *ad hoc* testing with *p*-values >0.8 between all groups shown in Figure 9A. Differential expression analysis between unsorted and sorted DPSCs treated with DMP1 generated 1934 differentially expressed genes (DEGs). Gene ontology biological process enrichment of the DEG profile demonstrated an enrichment of endothelial cell differentiation and function, angiogenic, cell morphogenesis and vasculature and tube development pathways. Genes upregulated in sorted DPSCs treated with DMP1 show presence of markers for cell morphogenesis (Figure 9B) and tube development (Figure 9C).

## Discussion

Blood supply is key in generating 3D tissues for regenerative medicine applications for cell survival, organ growth and integration with the host system (Aghazadeh et al., 2022). Therefore, development of strategies to generate functional ECs for cell-based therapeutic vascularization is a fundamental aspect of tissue engineering.

Several types of stem cell have been investigated for transformation into endothelial lineages (Jang et al., 2019). Of these, DPSCs are well suited for transformation into ECs and stimulating vasculogenesis. They are highly proliferative and demonstrate multipotent differentiation capabilities into osteoblasts, chondrocytes, and adipocytes (Aydin and Şahin, 2019; Luke et al., 2020). DPSCs were shown to readily differentiate into vascular endothelial lineages in the presence of VEGFR-1 compared with other mesenchymal cells derived from bone marrow and adipose tissues (Bergamo et al., 2021; Li A. et al., 2023). Further evidence also shows that DPSCs could be primed by VEGF to develop into vascular ECs and were identified by strong VEGFR1 expression. This transformation was attributed to VEGF binding to VEGFR1 instead of VEGFR2 (Bergamo et al., 2021). Studies from other group have demonstrated that VEGFR2 knockdown in DPSCs led to downregulation of VEGF and VEGFA receptors, as well as a reduction in the *in vivo* activation of angiogenic factors and odontoblastic differentiation of DPSCs (Janebodin et al., 2013, 2021) Therefore, to induce the differentiation of DPSCs towards the endothelial cell lineage, a combination of growth factors and differentiation agents are required as external stimuli.

In *in vivo*, the ECM contributes to cell proliferation, migration, and differentiation by providing microenvironments containing biological cues. Mimicking the complexity of the ECM will lead to specific cues conducive to the lineage-specific differentiation of stem cells (Badylak et al., 2009), and scaffolds derived from the ECM provides closest composition to the native tissue. The ECM is a crucial acellular three-dimensional macromolecular network that plays a pivotal role in tissue structure and function. Consisting of various proteins such as collagen, fibronectin, laminin, and glycoproteins, the ECM not only offers mechanical support to cells but also influences their behavior and fate. Recent research has emphasized the multifaceted functions of the ECM, indicating its involvement in directing cellular self-differentiation and renewal processes (Oliveira et al., 2021). Immunostaining of HUVEC-ECM shows the presence of key ECM proteins such as collagen, laminin and fibronectin as shown in Figure 1A which could act as a cue/morphogen to alter the fate of DPSCs to endothelial lineage. The collagen and non-collagenous proteins in the HUVEC-ECM enabled the attachment of DPSCs and notably influenced the morphological changes as shown in Figure 1B. These results are in accordance with earlier observations reported using stem cells from exfoliated deciduous teeth (Gong et al., 2017).

Understanding the interaction between the ECM and stem cells is important to develop translational strategies for the use of HUVEC-ECM in tissue engineering applications. In fact, mineralization and angiogenesis are closely coupled processes during tooth development as shown by imaging and single cell RNA sequencing using a mouse model, suggesting that critical communications exit between mineralized tissue and vasculature (Matsubara et al., 2022).

DMP1 is a member of the small integrin-binding ligand N-linked glycoprotein (SIBLING) family, group of proteins found in the ECM of bone and tooth (Staines et al., 2012). Apart from its functions as a calcium phosphate mineral nucleator (He et al., 2003), it also functions as a morphogen and induces odontogenic differentiation of stem cells (Narayanan et al., 2001; Almushayt et al., 2006) and formation of calcified tissues. The addition of DMP1 to DPSCs seeded onto HUVEC-ECM significantly enhanced the differentiation of DPSCs to endothelial-like cells, resulting in an increased percentage of endothelial surface markers (CD31+/CD144+, **: *p* < 0.01). Furthermore, additional biochemical and molecular characterization supports the synergistic effect of DMP1 in promoting the differentiation of DPSCs towards the endothelial lineage. These findings illustrate that stimulating DPSCs with DMP1 in conjunction with HUVEC-ECM could present a robust strategy for the differentiation of DPSCs into endothelial lineage.

The characterization of the differentiated cells by flow cytometry reveals a higher percentage of CD31+/CD144+ ECs during the differentiation process spanning from 0 to 14 days. Consequently, DMP1 stimulation proved to be essential in providing cues that significantly enhanced the transformation process. The resulting ECs displayed distinct morphological changes resembling typical ECs. The functional properties assessment of the converted DPSCs-derived endothelial-like cells was conducted using a range of endothelial cell functional assays, including the tube formation assay and spheroid sprout assay. The differentiated ECs formed tube-like structures and capillaries, with observable angiogenic sprouts and upregulation of several angiogenic markers. Additionally, the tube formation assay demonstrated longer tubes with increased branching and looping in DPSC-derived endothelial-like cells. In contrast, DPSCs without DMP1 stimulation exhibited a reduced ability to form capillary-like structures. Findings from this functional study revealed a noteworthy increase in tubulogenesis and sprouting of the DPSC-derived endothelial-like cells with the supplementation of DMP1 during the transformation of DPSCs. In addition DMP1 was essential to form a tubular network, which remained stable even after 24 h, and to spread and attach on Matrigel (Pirotte et al., 2011). Taken together, these findings collectively suggest that DMP1 has the potential to function as a signaling molecule promoting the transformation of DPSCs into vasculogenic ECs. HUVEC ECM contributes to the organization and stability of developing endothelial tubular networks. Consequently, our findings indicate that the interaction between DPSCs and DMP1, combined with the cues from the HUVEC matrix, may be accountable for activating various signaling pathways that induce their differentiation into ECs, accompanied by alterations in cell shape and morphology.

Integrins establish dynamic connections between the intracellular actin cytoskeleton and the ECM, playing a critical role in transmitting bidirectional signals across the plasma membrane (Schwartz, 2010). Specifically, interactions between the ECM and α6β4 a laminin-binding integrin plays a crucial role in the sprouting of ECs from capillaries and in angiogenesis. αVβ3 notably, the outside-in signals mediated by integrin work in conjunction with other growth factor receptors facilitate cell proliferation and motility (Li S. et al., 2023; Pang et al., 2023). This outside-in signaling pathway involves a ligand binding to the extracellular domain of active integrin, triggering a conformational change in the cytoplasmic tail. As a result, kinases and adaptor molecules in the cytosol are activated, contributing to downstream cellular processes (Morse et al., 2014). The findings presented in this manuscript indicate that the expression of α6β4 and αVβ3 integrins is higher when supplemented with DMP1, as evidenced by the results obtained from RT-PCR and Western blotting analyses. Moreover, it is worth noting that the expression of αVβ3 integrin is induced in microvascular ECs by VEGF-A (Liu et al., 2003). Thus, the expression pattern of integrin subunits further authenticates DPSCs differentiation towards endothelial-like cells. VEGFA is known to promote endothelial cell migration and proliferation, as well as functions in angiogenesis (Pauty et al., 2018; Apte et al., 2019). This intricate interplay underscores the multifaceted nature of the regulatory mechanisms governing endothelial cell behavior and angiogenesis.

VE-cadherin expression plays a pivotal role in DPSC capillary sprouting and establishes a continuous loop with the host vasculature (Sasaki et al., 2020). Results from this study further substantiate this concept by illustrating that DMP1 enhances VE-Cadherin expression at both the gene and protein levels. This enhancement likely contributes to the development of capillary sprouting by the converted ECs. Other proteins of interest that were upregulated with DMP1 stimulation were VEGFR2 and GRP78 (glucose-regulated protein 78) a member of the heat shock protein 70. We had demonstrated earlier that GRP78 functions as a receptor for DMP1 and facilitates its internalization (Ravindran et al., 2008). A previous study showed that in ECs, GRP78 was related to AKT-dependent angiogenesis. However, the mechanism related to GRP78 activation of AKT remains unknown (Raiter et al., 2010).

RNA sequencing data emphasizes the remarkable similarity in gene expression signature between the sorted DPSCs treated with DMP1 and the positive control HUVECs. This discovery underscores the potential influence of DMP1 treatment on the gene expression patterns of DPSCs, indicating a significant resemblance to the gene expression signature observed in HUVECs. These findings could lead to a comprehensive conception of the molecular mechanisms underlying the effect of DMP1 treatment on DPSCs, particularly in the context of transformation to ECs.

**FIGURE 10:**
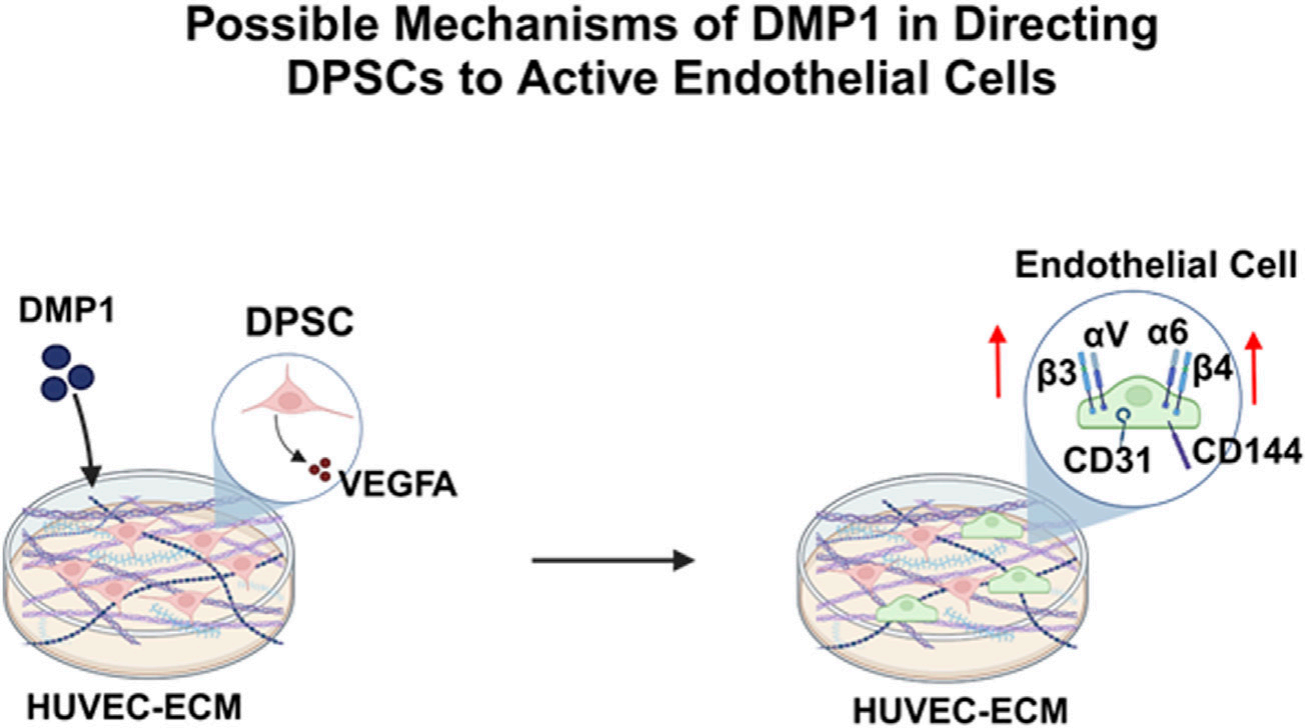
A schematic model depicting the DMP1 signaling pathway, resulting in the activation of VEGFA, Integrins (α6β4, αvβ3) and specific angiogenic proteins (VE-Cadherin, CD31), that play a crucial role in promoting the differentiation of DPSCs into endothelial-like cells using HUVEC-ECM scaffold.

In summary, we have demonstrated the ability of the HUVEC-ECM scaffold along with DMP1 stimulation to drive endothelial lineage-specific differentiation of DPSCs. Highlights from this study indicate that DMP1 stimulation influenced the expression of endothelial cell markers, VEGF, integrins and its downstream effectors (Figure 10). Our future efforts will be directed toward understanding this process in a more detailed manner to better understand the functional complexities of HUVEC-ECM and DMP1-mediated signaling.

## Conclusion

In this study, we have developed a methodology for robust lineage-specific differentiation of DPSCs into VE-Cadherin^+^/CD31^+^cells. These cells showed endothelial phenotype by their morphology, expression of EC markers at the mRNA and protein levels and expression of EC-specific integrins. Functional properties of the differentiated cells showed their ability to form vascular sprouts, tube-formation and vascular network. Overall, this study strengthens the utility of DPSCs for vasculogenesis by utilizing HUVEC-ECM scaffold and DMP1.

## Data Availability

The datasets presented in this study can be found in online repositories. The names of the repository/repositories and accession number(s) can be found below: https://www.ncbi.nlm.nih.gov/geo/,GSE266875.
